# Evaluating genotype imputation pipeline for ultra-low coverage ancient genomes

**DOI:** 10.1038/s41598-020-75387-w

**Published:** 2020-10-29

**Authors:** Ruoyun Hui, Eugenia D’Atanasio, Lara M. Cassidy, Christiana L. Scheib, Toomas Kivisild

**Affiliations:** 1 McDonald Institute for Archaeological Research, University of Cambridge, Cambridge, UK; 2 Department of Human Genetics, Katholieke Universiteit Leuven, Herestraat 49 - box 602, 3000 Leuven, Belgium; 3 Istituto di Biologia e Patologia Molecolari, Consiglio Nazionale delle Ricerche, Rome, Italy; 4 Smurfit Institute of Genetics, Trinity College Dublin, Dublin, Ireland; 5 Estonian Biocentre, Institute of Genomics, University of Tartu, Tartu, Estonia; 6 St John’s College, St John’s Street, Cambridge, CB2 1TP UK

**Keywords:** Anthropology, Archaeology, Evolutionary genetics, Population genetics

## Abstract

Although ancient DNA data have become increasingly more important in studies about past populations, it is often not feasible or practical to obtain high coverage genomes from poorly preserved samples. While methods of accurate genotype imputation from > 1 × coverage data have recently become a routine, a large proportion of ancient samples remain unusable for downstream analyses due to their low coverage. Here, we evaluate a two-step pipeline for the imputation of common variants in ancient genomes at 0.05–1 × coverage. We use the genotype likelihood input mode in Beagle and filter for confident genotypes as the input to impute missing genotypes. This procedure, when tested on ancient genomes, outperforms a single-step imputation from genotype likelihoods, suggesting that current genotype callers do not fully account for errors in ancient sequences and additional quality controls can be beneficial. We compared the effect of various genotype likelihood calling methods, post-calling, pre-imputation and post-imputation filters, different reference panels, as well as different imputation tools. In a Neolithic Hungarian genome, we obtain ~ 90% imputation accuracy for heterozygous common variants at coverage 0.05 × and > 97% accuracy at coverage 0.5 ×. We show that imputation can mitigate, though not eliminate reference bias in ultra-low coverage ancient genomes.

## Introduction

Over the past decade, the development of high-throughput sequencing methods has prompted an exponential increase in genome-wide sequences retrieved from ancient human remains; however, poor sample preservation often leads to low endogenous human DNA contents, thus a large body of ancient DNA (aDNA) samples remain sequenced at low coverage (1 × or less), leaving only a small proportion of individuals with high enough coverage to confidently call and phase diploid genotypes^1^.

Genotype imputation uses local linkage patterns to infer unknown genotypes in target samples from known genotypes, usually with the help of a reference panel of phased haplotypes. It has been routinely used on modern samples to extend SNPs genotyped on DNA microarrays to genome-wide variants for several applications^2,3^ There are many imputation tools available, most of which follow the Li and Stephens model^4^ to identify shared haplotypes between samples^2,3^. The accuracy and efficiency of the imputation algorithms have greatly improved over time; more recent tools such as Beagle 5 and IMPUTE 5 are scaled to work with reference panels containing millions of individuals^5,6^.

Compared to SNP genotyping, low-coverage shotgun sequencing data present a different challenge because we are not certain about any genotypes. One solution is to use a probabilistic measurement of the genotypes in the form of genotype probabilities or genotype likelihoods. Imputation tools that accept probabilistic genotype input include Beagle (≤ 4.1)^7^, IMPUTE 2^8^, and GLIMPSE^9^. Up until Beagle 4.0, the algorithm can produce genotypes for all sites in the reference panel in one step similar to IMPUTE 2 and GLIMPSE; in Beagle 4.1, the genotype likelihood mode only updates sites in the input file, requiring another step in the genotype mode to impute the missing genotypes.

Previous aDNA studies have used Beagle 4.0 to impute low-coverage ancient individuals using a one-step pipeline based on genotype likelihoods (detailed below)^4^, reaching accuracies above 99% for samples with a coverage as low as 1 ×^10–14^. Imputed data have been used to infer demographic history, genetic kinship and phenotypes, thus increasing the amount of information that can be obtained from archaeological samples and allowing ancient population-scale diploid studies. However, most aDNA sequences generated by shotgun sequencing do not reach the 1 × coverage threshold. aDNA also suffers from contamination and post-mortem damage that may not be fully accounted for in genotype likelihood models designed for modern DNA.

Here we break down the one-step pipeline, by first updating genotype likelihoods to genotype probabilities with the help of a reference panel and then filtering for highly confident genotypes. Treated as observed, these genotypes are then fed into standard imputation tools to estimate unknown genotypes. This procedure offers the flexibility to choose filters that are more stringent or more suitable for aDNA. Additionally, it allows us to benefit from recent imputation algorithms that are more efficient and accurate but mostly do not accept genotype likelihood input. We show that this pipeline can further improve imputation accuracy in ultra-low coverage (< 1 ×) ancient individuals.

## Method

### Imputation pipeline

**Figure 1:**
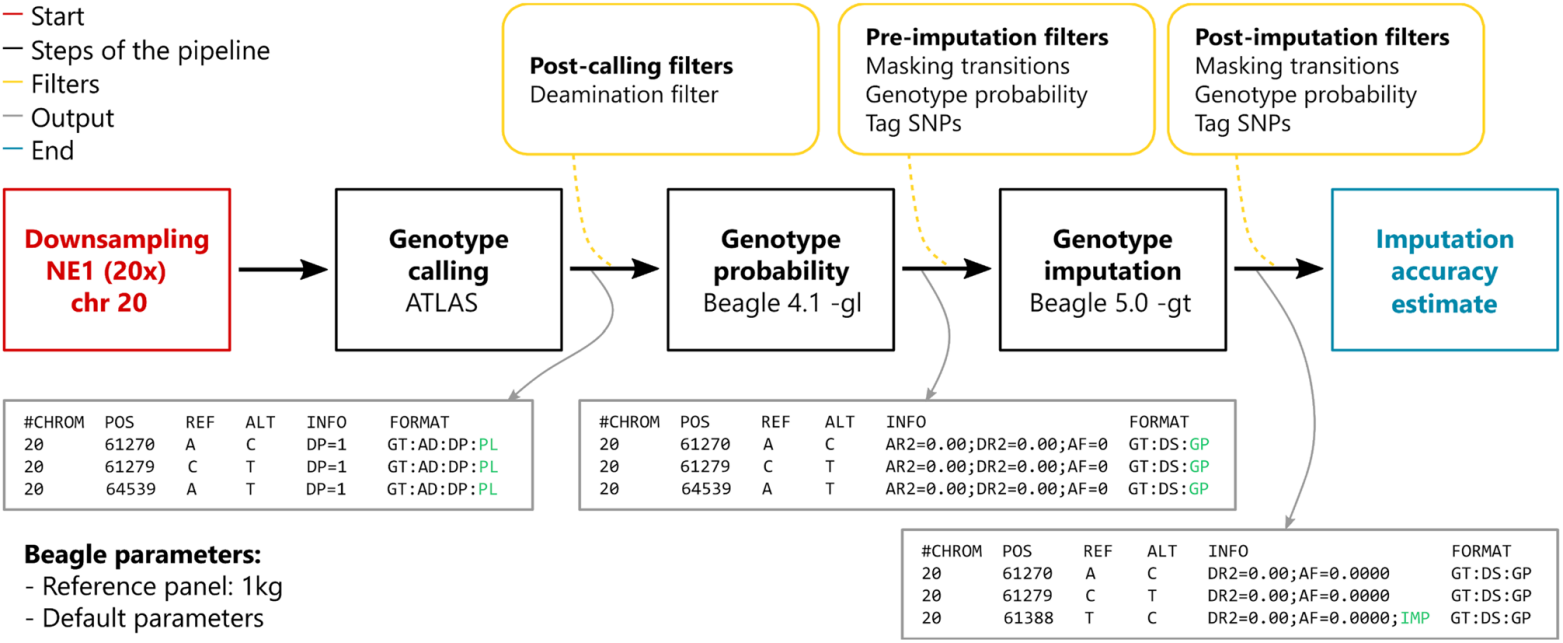
Schematic representation of the imputation pipeline. The input and output of the starting down-sampling step are both alignment files in BAM format. The output of each step of the pipeline (genotype calling, genotype probability update and genotype imputation) is a VCF file. In the output boxes, data fields that are updated and necessary in the following step of the pipeline are highlighted in green (1KG: 1000 Genomes).

The two-step imputation pipeline and all parameters that were tested within it are summarised in Fig. 1.

The two-step pipeline utilises previously described genotype calling^15–17^ and imputation^5,7^ methods to infer diploid genotypes from mapped read data (BAM file format) within a framework of several quality filters (Fig. 1 and Supplementary Material). First, genotype likelihoods are calculated by a genotype caller at variable positions in a reference panel. The imputation process then consists of two steps: the first step (GLIMPSE or -gl mode in Beagle 4.0/4.1) updates genotype likelihoods based on a reference panel; the second step then imputes missing genotypes from confident genotype calls, as determined by their genotype probability score^5,7^.

### Quality filters

We introduce optional quality control filters at three stages of the pipeline: after genotype likelihoods are called, we can use a post-calling filter to exclude the records at certain sites, e.g. removal of genotype likelihoods that could be the result of post-mortem damage; the pre-imputation filter removes low-confidence genotypes, which will be imputed back; the post-imputation filter excludes certain imputed genotypes from accuracy assessment and downstream analysis (Fig. 1).

Each filter aims to balance between the amount and the accuracy of genetic information passed onto the next step in the pipeline, although the optimal balance might differ across the stages. The user is free to design the filtering criteria at each stage. Here we considered several filtering criteria. First, considering that *post-mortem* deamination frequently leads to C to T (and G to A) mutations in aDNA molecules, we tested a transversion-only filter to exclude all transitions pre- and/or post-imputation. In the post-calling stage this filter was restricted to only exclude C to T and G to A mutations but retain transitions in the other direction, in order to preserve as much information as possible from low-coverage genomes. Most imputation tools report a measure of imputation uncertainty; in the case of Beagle, this takes the form of genotype probabilities (GP) corresponding to the genotypes being 0/0, 0/1 and 1/1. We can require the largest GP to exceed a threshold (e.g. 0.99) to enrich for confident genotypes in the pre- and post-imputation stages. Finally, because imputation relies on local linkage disequilibrium between variants, those in tight linkage with more neighbouring variants are expected to be easier to impute. We also tested pre- and post-imputation filters requiring at least 20 tag SNPs within 1000 kb of the variant (Supplementary Methods).

### Imputation accuracy

We evaluated the imputation accuracy by down-sampling a non-UDG treated 20 × Neolithic Hungarian genome (NE1)^10^ to lower coverages (from 0.05 × to 2 ×) and comparing the genotypes produced by the pipeline to those called in the original 20 × genome (Supplementary Methods). For example, the accuracy for heterozygous sites is calculated as the proportion of sites that are imputed as heterozygous given that they are confidently called as heterozygous in the 20 × genome. We report two metrics: imputation accuracy is calculated only in sites where the genotype is present in the imputed genome and confidently called in the 20 × genome; but since some post-imputation filters increase imputation accuracy at the cost of excluding many sites, we also report the raw number of correctly imputed sites and the proportion to sites called in the 20 × genome. A total of 2.1e7 confidently called sites (among which 1.8e6 are heterozygous) in the original genome were used as the gold standard to evaluate various settings in the pipeline. Some evaluations across different coverages are performed only on chromosome 20, where 4.7e6 sites (among which 4.0e5 are heterozygous) were confidently called in the original genome. Phased genomes (n = 2504) from the 1000 Genomes Project Phase 3^18^ (1KG) are used as the reference panel in our tests, unless otherwise noted.

## Results

### Default imputation pipeline

By default we used the following settings, which provide a good balance between imputation accuracy and the number of genotypes retained in the end: (a) genotype calling using ATLAS^17^ (maximum likelihood mode); (b) no post-calling filter application; (c) genotype probability update using Beagle 4.1; (d) application of a GP pre-imputation filter: max(GP) ≥ 0.99; (e) genotype imputation of missing sites using Beagle 5.0; (f) application of a GP post-imputation filter: max(GP) ≥ 0.99. More details can be found in the Supplementary Methods and the online repository (https://github.com/ryhui/imputation-pipeline).

Table 1 summarises the performance of our default imputation pipeline as evaluated on chromosome 20 of NE1 down-sampled to 0.05–2 ×. In addition to the overall measurements, we calculated accuracies separately for sites that are called as homozygous reference (0/0), homozygous alternative (1/1), and heterozygous (0/1) in the full-coverage genome and further partition them according to the minor allele frequency (MAF) in the 1KG reference panel. The accuracy at homozygous sites is much higher than at heterozygous sites, exceeding 0.97 at all coverages tested and even 0.99 above 0.5 × (Table S1). We were able to recover more than half of the variants in NE1 at > 0.95 accuracy from coverage as low as 0.05 × (Tables S1 and S2). The overall accuracy will be affected by the heterozygosity, which varies between genomes. We therefore limit our subsequent discussion to heterozygous sites to provide a lower bound of imputation performance.

**Table 1:** Imputation accuracy of the default pipeline across all coverages in NE1 chromosome 20.

Minor allele frequency bins	Coverages						
	0.05 ×	0.1 ×	0.5 ×	0.75 ×	1 ×	1.5 ×	2 ×
**Het accuracy**							
0.001–0.01	0.490	0.614	0.720	0.731	0.743	0.753	0.765
0.01–0.05	0.574	0.712	0.835	0.844	0.849	0.858	0.869
0.05–0.1	0.837	0.881	0.944	0.947	0.949	0.951	0.959
0.1–0.3	0.871	0.922	0.972	0.972	0.977	0.975	0.979
> 0.3	0.923	0.955	0.984	0.982	0.984	0.982	0.985
Common variants (MAF ≥ 0.05)	0.891	0.933	0.975	0.974	0.977	0.976	0.980
**All variant types accuracy**							
0.001–0.01	0.994	0.994	0.994	0.994	0.994	0.994	0.994
0.01–0.05	0.991	0.991	0.993	0.993	0.993	0.993	0.994
0.05–0.1	0.983	0.984	0.991	0.992	0.992	0.992	0.993
0.1–0.3	0.967	0.974	0.988	0.989	0.990	0.989	0.991
> 0.3	0.961	0.966	0.986	0.987	0.988	0.988	0.989
Common variants (≥ 0.05)	0.970	0.974	0.988	0.989	0.990	0.989	0.991

Accuracies are also shown for various MAF bins and genotypes in the full-coverage genome. Accuracies for homozygous sites are presented in Table S1; the actual numbers of correctly imputed sites are presented in Table S2.

**Figure 2:**
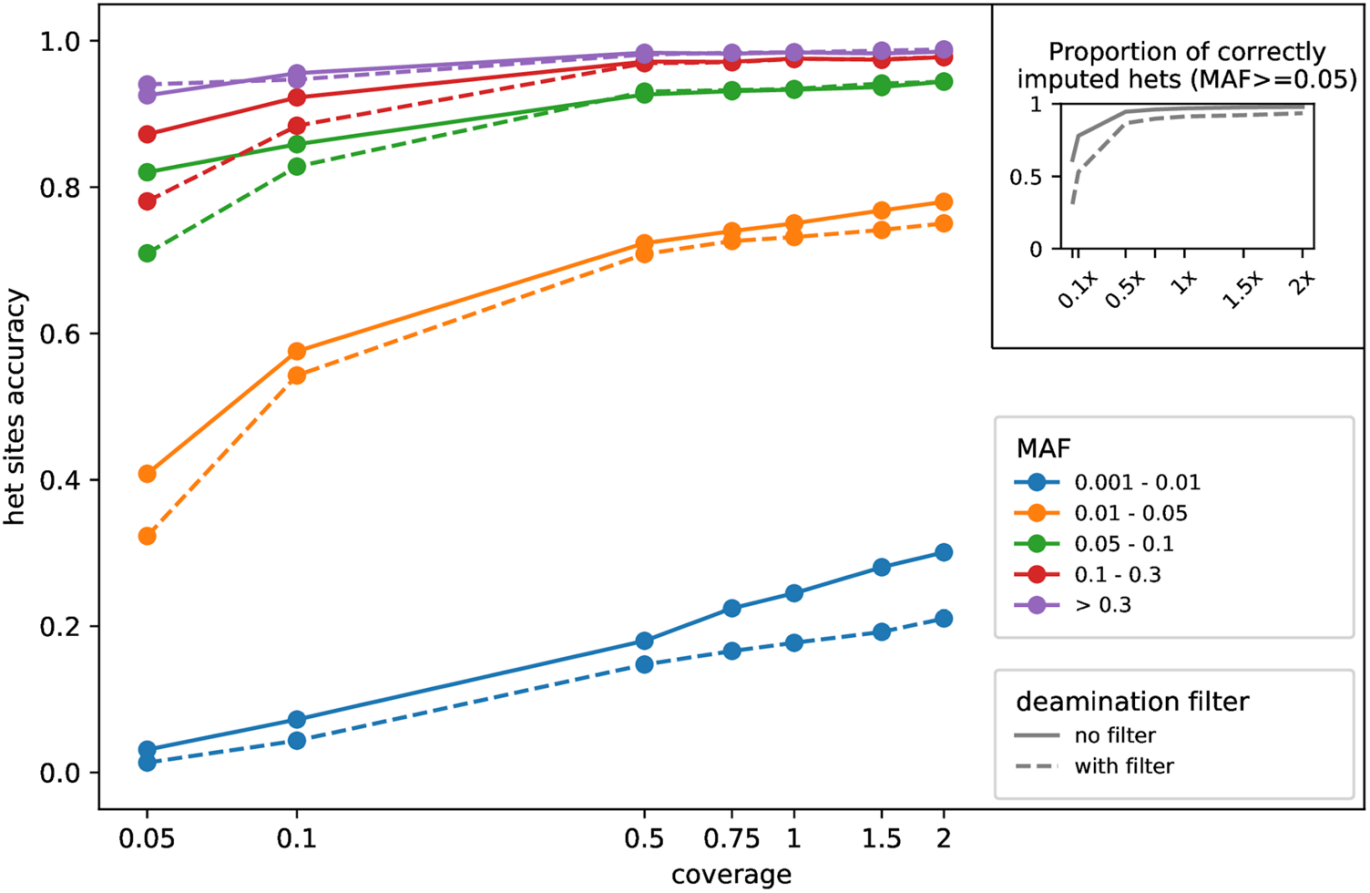
Imputation accuracy of heterozygous sites following the default pipeline evaluated by down-sampling NE1 chr20. The main figure shows the accuracy across coverages (on a log scale, X-axis), with and without the post-calling deamination filter. The inset on the top-right corner shows the proportion of heterozygous sites called in the original 20 × genome that are correctly imputed (i.e. not imputed as homozygous or failing the post-imputation filter).

In general, we observed that the accuracy at heterozygous sites depends heavily on variant frequencies, with the most common variants (MAF ≥ 0.3 in the reference panel) reaching 90% accuracy even at 0.05 ×, the lowest coverage tested (Table 1 and Fig. 2). On the other hand, rare variants (MAF < 0.05) remain difficult to impute even at 2 ×.

### Comparison with one-step imputing from genotype likelihoods

**Figure 3:**
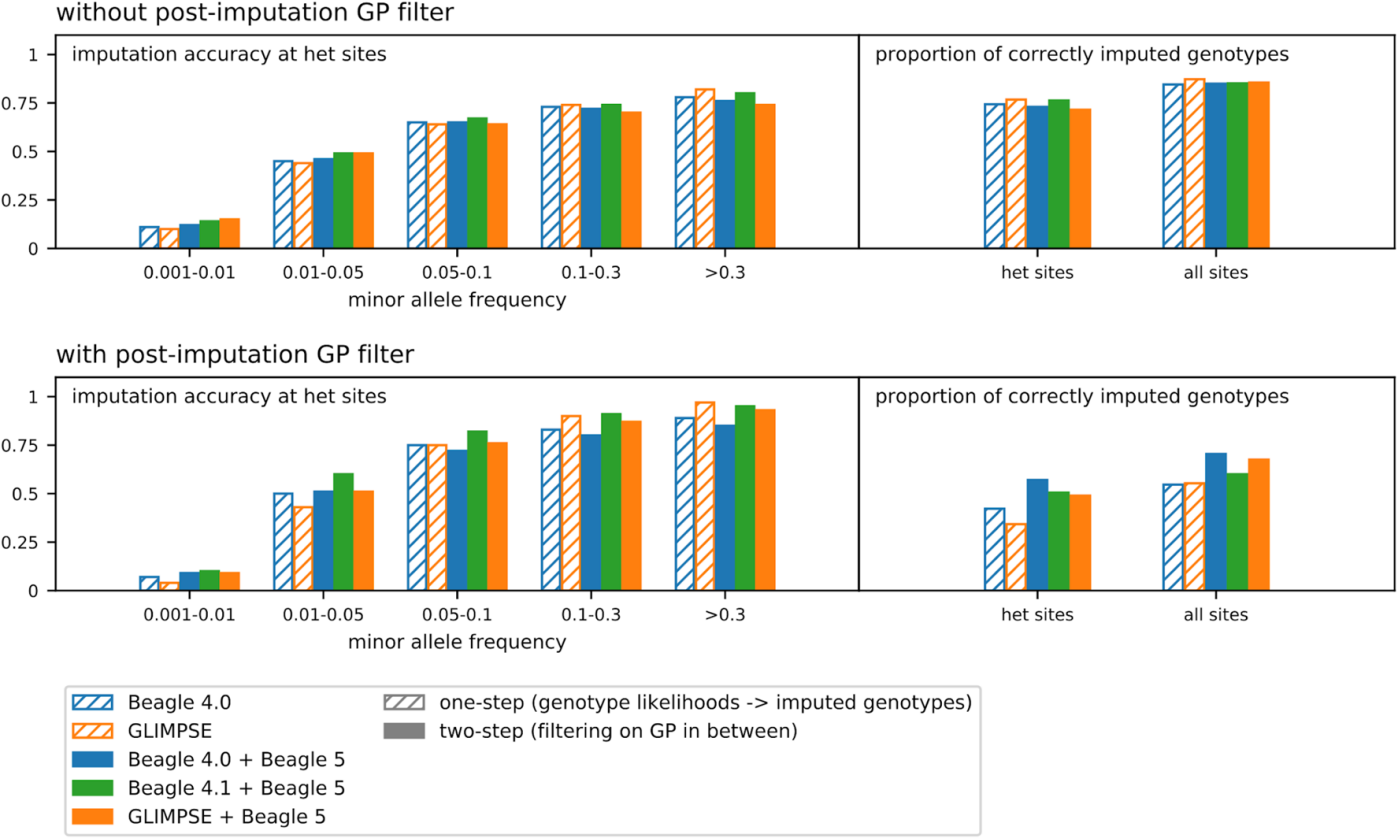
Comparing performance between one-step and two-step imputation pipelines. Two-step pipelines have a pre-imputation filter applied: max(GP) ≥ 0.99 for Beagle 4.0 + Beagle 5 and Beagle 4.1 + Beagle 5; max(GP) ≥ 0.9 for GLIMPSE + Beagle 5. In the lower panel, post-imputation GP filters are max(GP) ≥ 0.9 for GLIMPSE and max(GP) ≥ 0.99 for all the others. We used a more relaxed cutoff for GPs generated by GLIMPSE because these appear more conservative than GPs generated by Beagle 4 and 5 (Table S3).

Previous aDNA studies have used Beagle 4.0 to impute ancient genomes^10–13^, which accepts genotype likelihood input to estimate genotypes at all sites in the reference panel in a single step. Another imputation tool recently developed for low-coverage sequencing data, GLIMPSE, functions in a similar way^9^. We compared the performance of these one-step procedures to our two-step pipeline (Fig. 3, Table S3). Although the overall accuracy is similar without any post-imputation filters, the imputation algorithm reports more differential genotype probabilities when uncertain genotypes are excluded prior to imputation in our proposed two-step pipeline. As a result, more variants were obtained at a lower error rate when we apply the “max(GP) ≥ 0.99” filter after imputation (Fig. 3 and Table S3) (the same trend that we observe when evaluating the effect of other pre-imputation filters, described in a later section). We note that the two-step pipeline combining Beagle 4.0 and Beagle 5 does not improve imputation accuracy in common variants compared to Beagle 4.0 alone when the post-imputation filter is applied. Hence, part of the superior performance in the Beagle 4.1 + Beagle 5 pipeline, which we use as the default, can be attributed to the improved algorithm in Beagle 4.1 over Beagle 4.0.

We next explored the effect of various settings in the imputation pipeline, using our default setting as the baseline and changing one element at a time.

### Post-calling deamination filter

We tested a post-calling filter on all the coverages from 0.05 × to 2 ×, by masking out genotype likelihoods supporting C to T (G to A) mutations. We observed that this filter reduces the number of confidently imputed sites as well as accuracy at coverages below 0.1 × (Fig. 2; Table S4), suggesting that the loss of information outweighs the benefits from lower error rate. The difference in common variants becomes narrower above 0.5 ×, yet the deamination filter does not appear to convey any detectable advantage.

### Genotype callers

**Figure 4:**
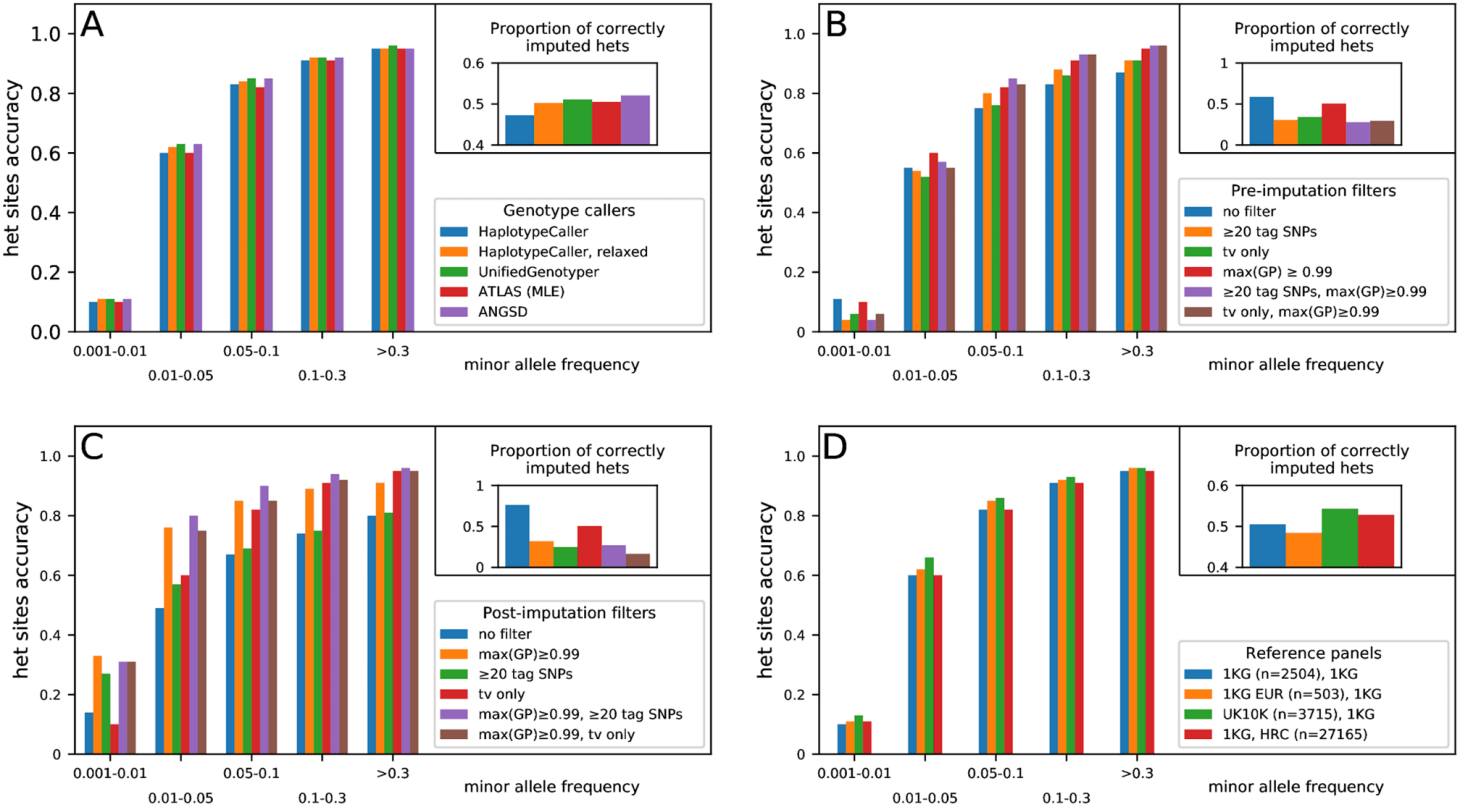
Effect of different settings on imputation accuracy evaluated by down-sampling NE1. (**A**) Performance using different genotype callers in a 0.05 × coverage genome; (**B**) Effect of pre-imputation filters in a 0.05 × coverage genome; (**C**) Effect of post-imputation filters at in a 0.05 × coverage genome; (**D**) Performance using different reference panels during the genotype probability update and imputation steps in a 0.05 × coverage genome. The inset on the top-right corner shows the proportion of heterozygous sites called in the original 20 × genome that are correctly imputed (i.e. not imputed as homozygous or failing the post-imputation filter). tv: transversion.

We then tested all the other parameters only on the 0.05 × down-sampled version of NE1 (Table S5). Comparison between genotype callers suggests that the performance of GATK^15^ HaplotypeCaller could be improved by increasing its sensitivity (--min-pruning = 1, --min-dangling-branch-length = 1). GATK UnifiedGenotyper, ATLAS, GATK HaplotypeCaller with relaxed settings, and ANGSD^16^ show comparable performance in general (Fig. 4A; Table S5). We chose to use ATLAS in the default pipeline for its built-in capacity to calibrate for *post-mortem* damage without comparing with the reference genome, which has been shown to reduce reference bias in low coverage genomes^17^. Although since *post-mortem* damage recalibration is not applicable to very low-coverage genomes, we did not perform it in our test to obtain more conservative results.

### Pre-imputation filters

Among criteria we tested to filter for reliable genotypes before the imputation step, the most effective one is based on GPs produced by Beagle. More precisely, filtering for confident genotypes (e.g. max(GP) ≥ 0.99) before imputation does not always improve overall accuracy, but generates more discriminating GPs for imputed genotypes, which facilitates post-imputation filtering. On the other hand, applying other filtering criteria based on tag SNPs or only transversions performs worse than not applying pre-imputation filters at all (Fig. 4B; Table S5). Adding the transversion-only filter or the tag SNP filter on top of the GP filter increases slightly the accuracy in common variants (MAF ≥ 0.05) at the cost of rare variants (MAF < 0.05) but greatly reduces the total number of usable genotypes.

### Imputation tools

Many tools and services are available to impute missing genotypes from known ones. We compared other popular tools and services in addition to Beagle 5.0, including IMPUTE4^19^, the Sanger Imputation Server^20–22^, and the Michigan Imputation Server^23^ starting from the same filtered genotypes (Table S5). 1KG was chosen as the imputation reference panel to make results comparable, although both imputation servers also provide larger reference panels. We find that Beagle 5.0 achieved the highest accuracy among heterozygous sites, but IMPUTE4 produced more correct genotypes by raw numbers.

### Post-imputation filters

GPs, the number of linked SNPs and/or types of mutations (transition vs. transversion) can also be used as post-imputation filtering parameters to select a subset of more confident variants (Fig. 4C; Table S5). In our test on chromosome 20, the accuracy for heterozygous sites gets close to 99% at coverages as low as 0.1 × when we combine filters based on GPs (≥ 0.99), types of mutations (transversion only) and the number of tag SNPs (≥ 20) (Table S6). They represent ~ 8% of all heterozygous calls in the original 20 × genome, but nevertheless suffice for many downstream analyses that do not require dense genetic markers.

Because the combination of quality filters sometimes retains relatively few sites for evaluation, we also tested the default pipeline, the post-calling deamination filter and the pre-imputation tag SNP filter on repeated down-samplings of chromosome 20. The results confirm that even at 0.05 ×, the observed effect of quality filters is not a result of stochasticity (Table S7).

### Reference panels

Finally, we tested how the choice of reference panel affects imputation accuracy (Fig. 4D). Genotype likelihood input is not supported after Beagle 4.1^7^, which does not scale to reference panels larger than a few thousand genomes, depending on the density of input markers and window size. A reference panel similar to the target sample in the first Beagle step performs slightly better than a world-wide reference panel. In the imputation step, a larger reference panel has little impact on the accuracy after applying the GP filter, but increases the number of genotypes passing the filter (Table S5). Therefore, we suggest first selecting a small reference panel as similar as possible to the target samples when updating the genotype likelihoods then switching to a larger world-wide reference panel in the imputation step.

### Considerations in local imputation

For some applications, such as the analysis of Mendelian traits, only the genotype/haplotype information of a short chromosomal region is required. So, we tested our imputation pipeline on short regions (1–5 Mb) of chromosome 20 of the NE1 genome (Supplementary Material). We tested four regions of different size and the results are highly concordant (Table S8), suggesting that also a small region can be used as a target for local imputation allowing to save computation time without losing accuracy. Since the computational time to perform the local imputation is shorter compared to the global imputation, we used this approach on 10 Mb to test some parameters also useful for the global imputation, namely MAF or minor allele count (MAC) filters on the reference panel and length and overlap of the Beagle 4.1 sliding windows at the genotype likelihood update step of our two-step pipeline. As for the MAF or MAC filters, we observed that in the absence of filters does not improve the accuracy (Table S8). When filters are applied, more strict filtering criteria (i.e. MAF ≥ 0.01) increase the imputation accuracy. However, rare variants (MAF < 0.1) showed higher accuracy estimates when relaxed filters (MAC ≥ 5) are applied on the reference panel, suggesting that more information is required to impute them. Regarding the window parameters (length and overlap) of Beagle 4.1, we found that windows smaller than the default value should be preferred for low coverage genomes (Table S8). Results from replicates on the HLA locus on chromosome 6 (Table S9) are consistent with chromosome 20, suggesting that our findings are not specific to the chromosomal context.

### Biases towards the reference genome and the reference panel

Pervasive reference bias is a major concern in aDNA analysis^24^. f4 statistics show that the imputed NE1 genomes are less biased towards the human reference genome compared to pseudo-haploid genotypes called on the down-sampled genomes, although the bias is not eliminated, consistent with previous observations^12^ (Table S10). Besides the reference genome bias in variant calling, we also assessed the bias towards the reference panel after imputation by calculating f4(20 × genome, down-sampled or imputed genome; 1KG population, ancestral genome) and found that both the pseudo-haploid and imputed genomes show reduced affinities to all modern populations included in the reference panel compared to the original 20 × genome (Table S10). This can also be explained by the bias towards the reference genome: the reference allele is more likely to be ancestral than derived, hence the derived alleles shared between the original NE1 genome and modern populations are often lost after pseudo-haploid calling or imputation. In fact, the most significant changes are found in European populations that are closest to NE1. Nevertheless, the outgroup f3 statistics to reference populations are highly correlated (R^2^ > 0.98) between the 20x, down-sampled and imputed genomes (Table S11), indicating that imputation does not cause the genome to become closer to a subset of the reference panel.

## Discussion

The default settings in the two-step pipeline presented here are a recommendation for imputing ultra-low coverage (≤ 1 ×) genomes, in cases where most of the genetic ancestry components are expected to be represented in the reference panel. By adding pre- and post-imputation filters based on the maximum genotype probabilities, we were able to recover more genotypes at increased accuracy compared to directly imputing from genotype likelihoods input. Reducing the effect of post-mortem damage biochemically by UDG treatment or computationally during mapping^17^ is likely to further improve the performance.

Despite the promising results of our pipeline, we also must note some limitations. Since we focused on extremely low coverages, the strategy of starting from genotype likelihoods may not be optimal for higher coverages. We tested the pipeline on down-sampled copies of a 20 × ancient genome to ensure that we can obtain enough “true” genotypes to compare with the imputed genomes. Genomes at lower coverages might still allow a subset of variants to be called confidently, but these variants are also more likely to be covered by reads after down-sampling, which would bias our estimation of the proportion of correctly imputed sites. We would expect a higher imputation accuracy for Western European individuals after the Neolithic period, as they should be closer than the NE1 genome we tested to individuals in the reference panel. These results may not be transferable to other populations, dependent on their demographic history and representation in the reference panel. In particular, accurate imputation will be difficult in populations harbouring ancestry components that are extremely rare or absent in the present-day reference panels, such as the European early hunter gatherer ancestry in Europe^29^. It will be helpful to evaluate the pipeline using high-coverage ancient genomes from outside Europe when they become available.

We also have to note that the imputation accuracy is still low for rare variants (MAF < 0.05), which show only < 60% accuracy for heterozygous sites at coverage 0.05 × (i.e. compared to > 85% accuracy at coverage 2 × in the MAF category 0.01–0.05; SI Table S1), making their analysis challenging. Although imputation reduces the pervasive reference bias in down-sampled genomes, we should still be mindful about its presence (SI Table S8).

In conclusion, we recommend using imputed genomes for downstream analyses incorporating genome-wide markers segregating at relatively high frequencies. They have been used in population genetics studies for detecting runs of homozygosity and identical-by-descent segments, and local ancestry deconvolution^10–13,25^. It has also been shown that low-coverage sequencing combined with imputation is more cost-effective in GWAS and generating polygenic risk scores^26–28^. But caution should be taken against conclusions that are based on individual loci, or methods that are sensitive to rare variant genotype quality (e.g. methods based on sequential Markovian coalescence).

## Supplementary information

Supplementary Information.

## Data Availability

No new data were generated for this study. The previously published NE1 genome can be accessed from the NCBI Sequence Read Archive under the code SRX484078. Scripts to run the pipeline and evaluate imputation accuracy are available at https://github.com/ryhui/imputation-pipeline.
