# Supplementary material for: Evaluating genotype imputation pipeline for ultra-low coverage ancient genomes
Source: Sci Rep. 2020 Oct 29;10:18542. doi: 10.1038/s41598-020-75387-w (PMC7596702; doi:10.1038/s41598-020-75387-w)
Supplement: Supplementary file 1 — Supplementary Information. [file 41598_2020_75387_MOESM1_ESM.zip › SupplementaryMaterial_Imputation_rev1.pdf]

Evaluating imputation pipeline for ultra low coverage ancient genomes

Ruoyun Hui[1,^], Eugenia D'Atanasio[2,3,^], Lara M. Cassidy[4], Christiana L. Scheib[5,6], Toomas Kivisild[1,2,5,*]

[1] McDonald Institute for Archaeological Research, University of Cambridge, UK

[2] Department of Human Genetics, Katholieke Universiteit Leuven, Belgium

[3] Istituto di Biologia e Patologia Molecolari, Consiglio Nazionale delle Ricerche, Rome, Italy

[4] Smurfit Institute of Genetics, Trinity College Dublin, Ireland

[5] Estonian Biocentre, Institute of Genomics, University of Tartu, Estonia

[6] St John's College, St John's Street, Cambridge CB2 1TP, United Kingdom

^ These authors contributed equally to this work

*Correspondence: Toomas Kivisild, Department of Human Genetics, Herestraat 49 - box 602, B-3000 Leuven, Belgium. Email: toomas.kivisild@kuleuven.be

Supplementary Material

The sample

Raw data in the FASTQ format for the NE1 sample[1] were trimmed for residual adapter sequences using cutadapt v1.2.17 and mapped to hg19/GRCh37 with BWA version 0.7.59 using the non-default parameters *-l 16500, -n 0.02* and *-o 2*. Reads were sorted, filtered for a mapping quality (MQ) of 20 or above and PCR duplicates removed using Samtools v0.1.1910. Read groups were added and BAM files merged to sample level using Picard Tools v1.101 (http://broadinstitute.github.io/picard/). GenomeAnalysisTK v2.4-711 was used to locally realign reads. Two base pairs at both the 5' and 3' ends of reads had their qualities reduced to a PHRED score of 2 and reads below a length of 34bp were removed. Samtools was used to extract chromosome 20 and downsample the read coverage over this contig.

Genotype calling

We tested four genotype callers that produce genotype likelihoods from mapped reads, including GATK HaplotypeCaller, GATK UnifiedGenotyper, ATLAS and ANGSD.

GATK HaplotypeCaller (GATK v4.0.2.1)
Under default setting, we provided a list of variants in the imputation reference panel using the "--alleles" parameter, and set the program to output all given sites ("--genotyping-mode GENOTYPE_GIVEN_ALLELES --output-mode EMIT_ALL_SITES"). Under the relaxed setting, we also added "--min-pruning 1 --min-dangling-branch-length 1" to achieve more aggressive calling.

GATK UnifiedGenotyper (GATK v3.8.1.0)
Similar to GATK HaplotypeCaller, we set the program to output information at all polymorphic sites in the imputation reference panel and kept other parameters as default.

ATLAS (v0.9)
We used the maximum likelihood caller ("task=call method=MLE") and set the output VCF to include the PL field ("formatFields=GT,PL") at polymorphic sites in the imputation reference panel (setting "sites").

ANGSD (v0.921)
The SAMtools-like model ("-GL 1 -doPost 1") in ANGSD was used to produce genotype likelihoods at polymorphic sites in the imputation reference panel (passed through "-sites"), assuming known major/minor alleles ("-doMajorMinor 3").

Post-calling filter
When the deamination filter is applied, we removed records where the genotype with one or both alleles resulting from deamination (C->T or G->A) has the highest genotype likelihood among the three genotypes considered (the script is available at https://github.com/ryhui/imputation-pipeline). As a consequence, genotypes at these sites will be treated as missing and estimated during imputation.

Imputation
Genetic maps and reference panels
Except for the evaluation comparing reference panels (Figure 2E), we used 2,504 genomes from the 1000 Genomes Project Phase 3 (1KG)[2] as the reference panel in both Beagle steps. We used the version downloaded from the Beagle website (http://bochet.gcc.biostat.washington.edu/beagle/1000_Genomes_phase3_v5a/), which has been filtered to exclude structural variants and variants with MAC < 5. The human genetic maps on GRCh37 were also obtained from the same website (http://bochet.gcc.biostat.washington.edu/beagle/genetic_maps/).
The Human Reference Consortium panel[3] was obtained through the Europe Genome-phenome Archive (https://www.ebi.ac.uk/ega/studies/EGAS00001001710), which includes genomes from the UK10K project[4]. We also filtered both panels to exclude sites with MAC < 5.

Genotype likelihood update
We ran Beagle 4.1[5] with genotype likelihoods as input ("gl" parameter), and required the output to include genotype probabilities to facilitate pre-imputation filtering ("gprobs=true"). Beagle 4.1 uses the number of variants instead of genetic distance to specify window size. In order to maintain comparable window sizes for genomes down-sampled to different coverages, we used the total number of variants in the target divided by 15 as the window size. Since the total length of chromosome 20 is around 108 cM, this would exceed the recommended 5 cM minimum.
With GLIMPSE[6], we used 2Mbp windows with a buffer size of 200kbp (GLIMPSE_chunk --window-size 2000000 --buffer-size 200000). By default GLIMPSE does not impute variants not present in the input file, so we filled in uninformative PLs (0,0,0) at variants not covered by any sequencing reads.

Genotype imputation

After optional pre-imputation filtering, the genotypes were used as the input to Beagle 5.0[7] ("gt" parameter). Similarly we specified the output to include genotype probabilities ("gp=true"). Window and overlapping sizes were left as default (5 and 0.5 cM).

<u>Filter on the number of tag SNPs</u>

We used PLINK[8] (v1.9; http://pngu.mgh.harvard.edu/purcell/plink/) to search for tag SNPs of each target variant in the imputation reference panel. Tag SNPs were required to be located within 1000 kb from the target variant, and have an allele count squared correlation above 0.8 ("--show-tags all --tag-kb 1000 --tag-r2 0.8"). The filter then retained only variants with at least 20 tag SNPs. We tested the tag SNP filter both before and after the imputation step.

<u>Other pre- and post-imputation filters</u>

For both the pre- and post-imputation filters based on the genotype probability, we used bcftools[9] to keep only the variants with the highest GP ≥ 0.99. For the pre- and post-imputation "transversions only" filters, we removed records in the VCF files where the two alleles are C and T or G and A.

<u>Replications</u>

We ran our default pipeline on chromosome 6, using the same parameters explained above. To test the reproducibility of our results, we also ran four replications on chromosome 20. First, we produced additional four 0.05× versions of NE1 by downsampling it using different seed numbers (from 1 to 4) in the "samtools view -s" option[9]. We then replicated three tests: 1) our default pipeline; 2) our pipeline with the post-calling deamination filter; 3) our pipeline with the additional pre-imputation filter based on the tag SNPs.

Local imputation

<u>Imputation pipeline</u>

We used our default pipeline to impute a region of about 10 Mb in the chromosome 20 (chr20:27665748-38218090), selected by adding flanking wings of 5 Mb to a core region of about 500 kb (chr20:32665748-33218090). This core region was chosen because it includes three SNPs of the HIrisPlex-S kit for the phenotype prediction[10], which is one of the applications of our imputation approach[11,12]. All the tests have been run only on the 0.05x downsampled version of NE1.

<u>Size of the locally imputed region</u>

We tested three additional different sizes for the local imputation, in order to reach a compromise between speed and accuracy: 5 Mb, 2 Mb and 1Mb. All the regions have been selected by adding 2.5 Mb, 1 Mb and 0.5 Mb respectively to the core region of 500 kb.

<u>Parameters common between global and local imputation approach</u>

When the computation time was too long to test the parameters of interest with a global imputation approach, we used a local imputation approach.

First, we tested and compared three different filtering strategies on the variants in our reference panel: a) Default filters used for the global imputation; b) only biallelic variants with MAF < 1%; c) only biallelic SNPs, without any other filters based on MAF or MAC. The b) filters gave the best results and have been used for all the other tests, while keeping unaltered all the other options with the exception of the parameter under test.

Regarding the Beagle parameters for the -gl step, our estimated “window” and “overlap” values for the 0.05x version of NE1 were 2,000 and 200 variants respectively, corresponding to only one window for our region of 10 Mb. We also tested: a) window=50,000 and overlap=3,000, i.e. the default parameters according the Beagle 4.1 manual (one window); b) windows=1,000 and overlap=100 (two sliding windows); c) window=400 and overlap=40 (four sliding windows).

HLA locus

As for the global imputation approach, we tested our local imputation approach on the HLA locus on chromosome 6. We added 2 Mb at the HLA (1 Mb at each side), for a total of 6.9 Mb imputed. First, we ran our default pipeline and estimated its accuracy at this region. We then tested a post-imputation filter based on tag SNPs and, finally, we discarded the two 1 Mb flanking regions to assess the imputation accuracy specifically for the HLA locus. We compared the results of each local imputation test with the results for the same region extracted from the globally imputed chromosome 6, using the same parameters.

Imputation accuracy estimate

Genotype calling on the 20x NE1 genome

We used GATK haplotypecaller (v4.0.2.1) to call genotypes on the original ~20x genome at polymorphic sites in the imputation reference panel. We further filtered the genotypes for GQ ≥ 30 and DP ≥ 10 to exclude less confident calls. The remaining sites were treated as true genotypes to evaluate the accuracy of imputation. When testing the accuracy across various coverages, we only imputed chr20 to avoid long running time.

Calculating imputation accuracy

The output from imputation was compared to genotypes called at the full coverage genome, which were taken as the truth, using SnpSift[13] (version 4.1l). Imputation accuracy for heterozygous sites is reported in the form P(imputed genotype is “0/1” | true genotype is “0/1”), and likewise for homozygous reference and alternative sites. Since heterozygous sites turn out to be the most difficult to impute, we only report their accuracy in most tests as the lower bound for overall accuracy. When post-imputation filters were applied, sites not passing the filter were not included in the evaluation; we also report the total number of correctly imputed sites to reflect the trade-off. Minor allele frequencies were calculated from the 1KG reference panel. A Python script used to produce the accuracy tables is available at https://github.com/ryhui/imputation-pipeline).

References

1. Gamba, C. *et al.* Genome flux and stasis in a five millennium transect of European prehistory. *Nat. Commun.* **5**, 5257 (2014).
2. The 1000 Genomes Project Consortium. A global reference for human genetic variation. *Nature* **526**, 68–74 (2015).

3. McCarthy, S. *et al.* A reference panel of 64,976 haplotypes for genotype imputation. *Nat. Genet.* **48**, 1279–1283 (2016).
4. Walter, K. *et al.* The UK10K project identifies rare variants in health and disease. *Nature* **526**, 82–90 (2015).
5. Browning, B. L. & Browning, S. R. Genotype Imputation with Millions of Reference Samples. *Am. J. Hum. Genet.* **98**, 116–126 (2016).
6. Rubinacci, S., Ribeiro, D. M., Hofmeister, R. & Delaneau, O. Efficient phasing and imputation of low-coverage sequencing data using large reference panels. *bioRxiv* 2020.04.14.040329 (2020) doi:10.1101/2020.04.14.040329.
7. Browning, B. L., Zhou, Y. & Browning, S. R. A One-Penny Imputed Genome from Next-Generation Reference Panels. *Am. J. Hum. Genet.* **103**, 338–348 (2018).
8. Purcell, S. *et al.* PLINK: A Tool Set for Whole-Genome Association and Population-Based Linkage Analyses. *Am. J. Hum. Genet.* **81**, 559–575 (2007).
9. Li, H. *et al.* The Sequence Alignment/Map format and SAMtools. *Bioinformatics* **25**, 2078–2079 (2009).
10. Chaitanya, L. *et al.* The HIrisPlex-S system for eye, hair and skin colour prediction from DNA: Introduction and forensic developmental validation. *Forensic Sci. Int. Genet.* **35**, 123–135 (2018).
11. Saag, L. *et al.* The Arrival of Siberian Ancestry Connecting the Eastern Baltic to Uralic Speakers further East. *Curr. Biol.* **29**, 1701-1711.e16 (2019).
12. Scheib, C. L. *et al.* East Anglian early Neolithic monument burial linked to contemporary Megaliths. *Ann. Hum. Biol.* **46**, 145–149 (2019).
13. Cingolani, P. *et al.* A program for annotating and predicting the effects of single nucleotide polymorphisms, SnpEff. *Fly (Austin)* **6**, 80–92 (2012).
